# Identification of functional hubs and modules by converting interactome networks into hierarchical ordering of proteins

**DOI:** 10.1186/1471-2105-11-S3-S3

**Published:** 2010-04-29

**Authors:** Young-Rae Cho, Aidong Zhang

**Affiliations:** 1 Department of Computer Science Baylor University, Waco, TX 76798, USA; 2 Department of Computer Science and Engineering, State University of New York, Buffalo, NY 14260, USA

## Abstract

**Background:**

Protein-protein interactions play a key role in biological processes of proteins within a cell. Recent high-throughput techniques have generated protein-protein interaction data in a genome-scale. A wide range of computational approaches have been applied to interactome network analysis for uncovering functional organizations and pathways. However, they have been challenged because ofcomplex connectivity. It has been investigated that protein interaction networks are typically characterized by intrinsic topological features: high modularity and hub-oriented structure. Elucidating the structural roles of modules and hubs is a critical step in complex interactome network analysis.

**Results:**

We propose a novel approach to convert the complex structure of an interactome network into hierarchical ordering of proteins. This algorithm measures functional similarity between proteins based on the path strength model, and reveals a hub-oriented tree structure hidden in the complex network. We score hub confidence and identify functional modules in the tree structure of proteins, retrieved by our algorithm. Our experimental results in the yeast protein interactome network demonstrate that the selected hubs are essential proteins for performing functions. In network topology, they have a role in bridging different functional modules. Furthermore, our approach has high accuracy in identifying functional modules hierarchically distributed.

**Conclusions:**

Decomposing, converting, and synthesizing complex interaction networks are fundamental tasks for modeling their structural behaviors. In this study, we systematically analyzed complex interactome network structures for retrievingfunctional information. Unlike previous hierarchical clustering methods, this approach dynamically explores the hierarchical structure of proteins in a global view. It is well-applicable to the interactome networks in high-level organisms because of its efficiency and scalability.

## Background

Recent high-throughput experimental techniques, such as yeast two-hybrid system [1] and mass spectrometry [2], have made remarkable advances in identifying protein-protein interactions on a genome-wide scale. Since the evidence of protein-protein interactions provides insights into the underlying mechanisms of biological processes within a cell, the availability of a large amount interaction data has introduced a new paradigm towards functional characterization of proteins on a system level.

A protein interactome network is structured by the set of genome-wide protein-protein interactions determined in each organism. A wide range of computational approaches [3-6] have attempted to analyze the interaction networks effectively for the purpose of predicting protein function or detecting functional modules. However, unraveling the complex connectivity has been a critical challenge. The false positive interactions, which typically appear in high-throughput experimental data, and functionally inconsistent interacting pairs [7] have reinforced the complexity. Thus, refining the noisy data and restructuring the complex network into a well-organized data format should be crucial pre-processes to enhance the network analysis.

In recent years, it has been investigated that protein interaction networks are characterized by intrinsic features [8], such as high modularity and hub-oriented structure. A network comprises a collection of functional modules that are interpreted as sets of proteins participating in the same function [9]. In general, a module is considered as a sub-graph whose nodes are densely connected with each other and sparsely connected with the others. Density-based clustering methods have been proposed to seek densely connected sub-graphs using various density functions [10-13]. However, they are not able to capture the global patterns of functional organizations from protein interaction networks. Functional modules are typically organized in a recursive manner such that a module includes one or more sub-modules having more specific functions. Hierarchical clustering methods have thus been applied to the networks for finding functional organizations[14-17]. The bottom-up approaches iteratively merge nodes or sub-networks, whereas the top-down approaches recursively divide the network into sub-networks. However, as a critical drawback, they are typically sensitive to complex connectivity and noisy data.

Hubs in a scale-free network [8] play a central role in characterizing its structure. Intramodule hubs ('party' hubs) have high connectivity to the members in a module, and intermodule hubs ('date' hubs) bridge different modules [18]. Previous studies have observed that such hubs in protein interaction networks are essential in terms of functionality [19-22] and, in particular, intramodule hubs have low evolutionary rates [23,24]. The concepts of modules and hubs, extending from specific (local) to general (global), suggest the potential structure of a hierarchy that might be hidden in complex interaction networks. How can we then effectively extract the hierarchical structure of proteins from the complex network to reveal the global picture of functional organizations?

In this study, we present a novel method for restructuring a complex interactome network into a hierarchical data format in order to reveal functional hubs and organizations. Our algorithm uses a weighted interaction network as an input. Because the network includes a significant number of false positive connections, the reliability or intensity of interactions should be assessed and assigned into the edges as weights. For network restructuring, we design a path strength model which proposes the quantification of functional similarity between two proteins. The interactome network having complex connectivity is then dynamically converted into a hub-oriented tree structure by the definition of path-strength-based centrality. From the hierarchical structure, we score hub confidence for each node, and generate hierarchically organized clusters of proteins. Unlike degree as a local significance measure, the hub confidence estimates the global significance of nodes. It is thus capable of selecting hubs that are located in critical positions of the network. The experimental results demonstrate that the hubs with high confidence are essential for performing functions. In network topology, they mostly bridge different functional modules. Furthermore, our approach has higher accuracy in identifying functional modules than other hierarchical clustering methods.

## Methods

### Path strength model

The path strength *S* of a path *p* is defined as the product of the weighted probabilities that each node on* p* chooses its succeeding node. The weighted probability from a node* v_i_* to* v_j_* is the ratio of the weight between* v_i_* and* v_j_* to the sum of the weights between* v_i_* and its directly connected neighbors.(1)

where* p* = 〈*v*_0_, *v*_1_ …, *v*_n_〉*w*_*i*(*i*+1)_ denotes the weight of the edge between* v_i_* and *v*_(*i*+1)_, which is normalized into the range between 0 and 1.* d^wt^*(*v_i_*) represents the shape parameter that indicates the weighted degree of the node* v_i_*. The weighted degree of* v_i_* is the sum of the edge weights between* v_i_* and its neighbors. *λ* is the scale parameter which depends on the specific type, structure and properties of the input network. To make the problem simple, the scale parameter will be set by 1. Based on the assumption that the shape parameter does not force the starting and ending nodes of* p*, Formula 1 then becomes:(2)

The path strength of a path* p* thus has a positive relationship with the weights of the edges on* p*, and a negative relationship with the weighted degrees of the nodes on* p.* Formula 2 also implies that the path strength has an inverse relationship with the length of* p* because the weighted probability, *w*_*i*(*i*=1)_ / *d*^*wt*^(*v_i_*), is in the range between 0 and 1, inclusive. As the length of* p* increases, the product of the weighted probability decreases monotonically. In the same manner, as the average degree of the nodes on* p* increases, the path strength of* p* is likely to decrease.

Next, we formulate the functional similarity measurement between proteins based on the path strength model. The functional similarity* ℱ* between two proteins* a* and* b* in an interactome network is described as the maximum path strength between them.(3)

Since any node pair selected in a small world network [25] are directly or indirectly connected with a relatively small path length, the maximum path length between them is typically limited. However, Formula 3 still has a computational problem when it enumerates all possible paths between* a* and* b.* To solve the computational complexity, we restrict the maximal boundary of path length.

We define the *k*-length path strength* S_k_* as the maximum strength of all distinct paths with length* k* between* a* and* b.*(4)

Using a user-specified threshold* θ* to set the maximal boundary of *k*, the functional similarity* ℱ * between* a* and* b* is calculated by the maximum *k*-length path strength.(5)

where* l ≤ k ≤ l* +* θ* and* l* is the shortest path length between* a* and *b*. Based on the assumption that edge weights represent the likelihood of functional linkage of interacting protein pairs, Formula 5 measures the potential of functional association between two proteins, directly or indirectly connected in a protein interactome network.

### Network restructuring

Based on the path strength model and functional similarity measurement, we calculate the centrality for each node. The centrality* C* of a node* a* in a network* G*(*V, E*) is defined as the sum of the functional similarity scores between* a* and the other nodes in* V*.(6)

Formula 6 captures not only the nodes centrally located in the network but also the core proteins that functionally have a strong influence on the others. Our strategy for the network restructuring is to place the nodes with higher centrality on the upper level in a hierarchical tree structure. We define the set of ancestor nodes* T* of a node* a* as the nodes whose centrality is greater than the centrality of* a.*(7)

Among the nodes in* T*(*a*), the node that are functionally the most similar with* a* becomes the parent node *p*(*a*) of *a*.(8)

Selecting a parent node for each node by Formula 8 then efficiently constructs a hierarchical tree structure. The node having the highest centrality among all the nodes in the network has no parent and becomes the root node. This hierarchical structure is dynamically converted on network growth, depending on the distribution of the path-strength-based centrality of nodes.

### Identifying hubs and clustering proteins

We apply the tree structure of a protein interaction network to identify hub proteins. For each node* a,* we obtain the set of child nodes* D*(*a*) of* a.*(9)

We then recursively trace down the tree structure starting from* a* and combine every child node set to produce a set of all descendant nodes* L_a_* of *a*(10)

Using this Formula for all nodes except leaf nodes, we finally generate the list of descendant sets. According to connectivity patterns, hubs have been categorized into intramodule hubs and intermodule hubs, as discussed. We here provide a new definition of hubs, called structural hubs. These hubs are the core nodes to support the hierarchical structure representing a protein interactome network. The structural hubs are selected by estimation of hub confidence. The hub confidence* H* of a node* a* is calculated by the sum of the functional similarity scores between* a* and the members of* L_a_,* divided by the functional similarity score between* a* and its parent node. If* a* is the root node, we use the sum of the functional similarity scores between* a* and all the other nodes as its hub confidence.(11)

The hub confidence in Formula 11 quantifies how likely the node is to be a structural hub. Since an edge weight represents the functional consistency between two ending nodes, the structural hubs have a significant role in not only maintaining topology but also functionality.

We finally generate clusters as functional modules from the tree structure. We iteratively select a structural hub* a* with the highest hub confidence score and output* L_a_* as a cluster until the hub confidence of the selected node* a* reaches a user-specified threshold. The clusters are hierarchically arranged based on the positions of their hubs in the tree structure.

**Figure 1:**
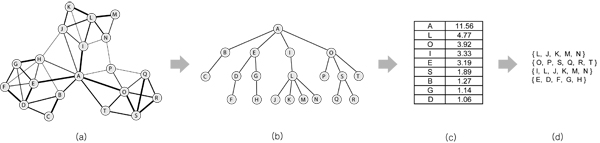
**The process of analyzing a protein interaction network by the network-conversion approach**. (a) A weighted interaction network is converted into (b) a hierarchical tree structure of proteins based on the path strength model and the functional similarity measurement. The edge weight was described as its thickness in (a). From the generated tree structure, (c) hub confidence is estimated for each node. From the selected structural hubs, (d) clusters as functional modules are identified.

The schematic view of our approach is illustrated in Figure 1 using a synthetic network with 20 nodes. In the input network 1 (a), the weight for each edge is described as its thickness. After the weighted network is restructured to a hierarchy 1 (b), the structural hubs are identified by scoring the hub confidence 1 (c), and the nodes are grouped to reveal hierarchically organized functional modules 1 (d). In the hierarchical structure, the depth of a node denotes the maximum path length from the node to a leaf node. The depth of a cluster is then defined as the maximum depth of nodes in the cluster, i.e., the maximum depth of nodes in a depth-*k* cluster is *k*. For example, in Figure 1 (b) and 1 (d),  { *D, F*} is a depth-1 cluster, and {*E, D, F, G, H*} is a depth-2 cluster. In typical, the functional module with a smaller depth is conceptually more specific and topologically denser in the network.

## Results and discussion

### Data sources

Currently, genome-wide protein-protein interaction data of several model organisms are publicly available in a number ofopen databases, for example, BioGRID [26], MIPS [27], DIP [28], MINT [29] and IntAct [30]. They have been mostly generated by high-throughput methods. However, because of unreliability of the high-throughput experimental data, we tested our algorithm using the core protein-protein interaction data of Saccharomyces cerevisiae from DIP, which were curated by other biological information such as protein sequences and expression profiles. They include total 2526 distinct proteins and 5725 interactions between them.

Since our approach requires a weighted interaction network as an input, we pre-computed the edge weight for each interaction in three different ways. First, we explored statistical significance of the alternative indirect connections for each pair of interacting proteins. Suppose* N*(*v_i_*) and* N*(*v_j_*) are the sets of directly connected neighboring nodes of* v_i_* and* v_j_*. To estimate the weight* w_i,j_* of the interaction between* v_i_* and* v_j_*, we used *p*—value from the hypergeometric distribution.(12)

Formula 12 indicates the probability that at least* |N*(*v_i_*) ∩* N*(*v_j_*)| proteins in* |N*(*v_j_*)| are included in* |N*(*v_i_*)| by random chance. In other words, it means the probability that two nodes* v_i_* and* v_j_* have alternative indirect paths with length-1. The weight* w_i,__j_* of the interaction between* v_i_* and* v_j_* can be then computed by(13)

Next, we applied gene co-expression profiles for interacting proteins. The gene expression data were obtained from SMD [31], and the coherence of expressions was calculated by the Pearson coefficient. Finally, we adopted annotations in the GO [32] database. The semantic similarity measure [5] was used to compute the functional similarity of each pair of interacting proteins.

**Figure 2:**
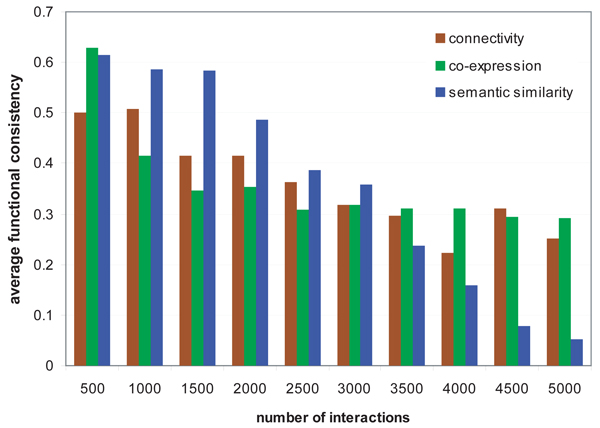
**Evaluation of interaction weights by functional consistency**. We computed edge weights of the yeast protein interaction network by statistical significance in connectivity, co-expression profiles, and semantic similarity of interacting protein pairs. We also measured functional consistency of each pair by the ratio of common functions. All weighting schemes have positive relationships between weights and functional consistency.

We assessed the edge weights in terms of functional consistency, the ratio of common functions to all distinct functions that the interacting proteins have. As the functional information of proteins, the annotation data on the 2nd-level functional categories from MIPS [27] were used. After arranging all interactions by their weights in descending order, we plotted the cumulative functional consistency with respect to the selected number of interactions in Figure 2. Comparing to the semantic similarity-based weighting scheme, the approach for statistical significance in connectivity did not select well both of the top 10% of the most functionally consistent interactions and the bottom 20% of the least consistent interactions. Weighting interactions by the co-expression-based method was also unsuccessful in the range between top 10% and 30%, and below 70%. However, in general, positive relationships are shown between functional consistency and the weights computed by these methods across all the range.

### Evaluation of path strength model

**Figure 3:**
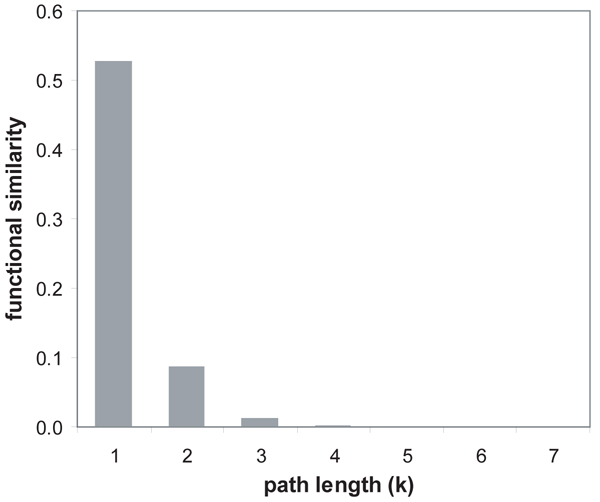
**The relationship between path length and functional similarity of randomly selected protein pairs.** Since the average functional similarity of the pairs having a path length greater than 3 is nearly 0, we computed path strength for each protein pair by limiting its length to (*l *+ 2) where *l* is the shortest path length of the pair.

We evaluated the effectiveness of our path strength model and functional similarity measurement in the weighted interaction network. For the calculation of functional similarity* ℱ *(*a,b*), we have to enumerate all *k*-length paths* S_k_*(*a, b*) between two proteins* a* and* b* for all possible *k*. However, the impact of* S_k_*(*a, b*) on *S*(*a, b*) in Formula 5 significantly decreases with the increment of *k*. In the experiment with randomly selected 10,000 protein pairs, the functional similarity rapidly decreases by the increment of path length, and is close to 0 with the path length of greater than 3, as shown in Figure 3. For efficient computation of functional similarity between* a* and* b,* we thus selected the maximum path strength by limiting the maximal k to (*l* + 2) where* l* is the shortest path length between them. In other words, we considered the paths between two nodes with length-* l*, (*l* + 1) and (*l* + 2).

**Figure 4:**
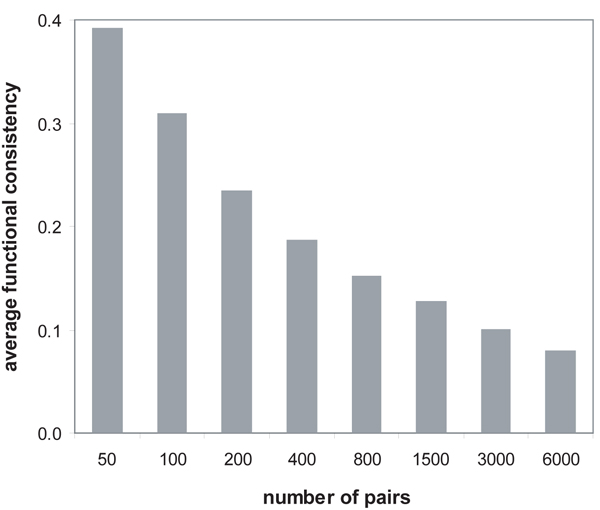
**Evaluation of the path strength model by functional consistency**. For the randomly selected 10,000 pairs arranged by their functional similarity from the highest, the cumulative functional consistency was calculated by the ratio of common functions. The average functional consistency between two proteins monotonically decreases as their functional similarity measured by our path strength model decreases.

We investigated the relationship between path strength and functional consistency to show whether a stronger path is still functionally more consistent. We first measured functional similarity for all possible pairs of proteins by Formula 5, selected 10,000 pairs randomly, and then computed the cumulative functional consistency of each selected pair in the same way described above. At this time, we used the weighted interaction networks produced by the third method integrated with GO annotations using the semantic similarity measure. In the arrangement of the selected protein pairs by their functional similarity in a descending order, the change of cumulative functional consistency was shown in Figure 3. The average functional consistency monotonically decreases as more pairs are included. It indicates that the pair having higher functional similarity on our path strength model are functionally more consistent. The average functional consistency in Figure 4 is lower than that in Figure 3 because all possible paths regardless of their path length were considered in Figure 4, whereas only length-1 paths (i.e., interacting proteins) were tested in Figure 3. However, the average functional consistency in Figure 4 is not very low because any two proteins are connected with each other in a few steps in a typical interaction network characterized by the small-world property [25]. The results in Figure 3 and 4 signify that our model is correctly designed to measure functional similarity between two proteins through network connectivity.

### Topological significance of structural hubs

We implemented the conversion of the weighted interaction network to a hierarchical tree structure by Formula 8. We then identified the structural hub proteins based on their hub confidence scores in Formula 11. To make topological assessment of the structural hubs, we tested network vulnerability on random and hub attacks. It has been known that typical scale-free networks are robust on random attacks, but vulnerable on targeted attacks to the hubs. For this experiment, we observed the fractions of the largest component when we repeatedly disrupted a randomly selected node, a hub with the highest degree and a structural hub with the highest hub confidence score, respectively.

**Figure 5:**
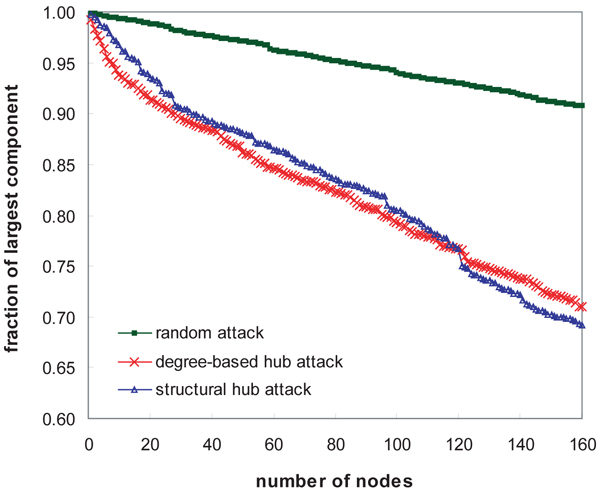
**Assessment of topological significance of the structural hubs by network vulnerability**. We repeatedly disrupted a randomly selected node, a hub with the highest degree and a structural hub with the highest hub confidence score, respectively, and monitored the fraction of the largest component connected. The network was more vulnerable on the degree-based hub attacks and structural hub attacks than the random attacks.

Figure 5 shows the comparative result of network vulnerability. Because all nodes in the network are directly or indirectly connected with each other, the fraction of the largest component is 1 before the node removal. Removing hubs decreases the fraction more rapidly than removing random nodes. In Figure 5, we can observe the remarkable difference of the deceasing rates between hub attacks and random attacks. In comparison of structural hubs and degree-based hubs, the network was more susceptible to the degree-based hub attacks when top 10 hubs were removed. However, after removing 120 hubs, the structural hub attacks were more destructive. In further experiments, we compared the hub confidence measure in Formula 11 with node degree. Since all degree-1 nodes are the leaf nodes in the tree structure, their hub confidence is 0. For the nodes whose degree is greater than 1, the hub confidence has a monotonic increase by the increment of their degree.

Overall, a protein interaction network is more vulnerable on structural hub attacks than random attacks. It is noticeable that the hub confidence measure is effective at selecting topologically significant hub proteins in complex networks. In general, hub confidence has a positive relationship with node degree. However, some low-degree structural hubs with high hub confidence can be detected by our algorithm. Whereas degree is a factor for local significance of nodes in network topology, the hub confidence formula measures the global significance of nodes to select hubs in the hierarchical structure.

### Biological essentiality of hub proteins

**Figure 6:**
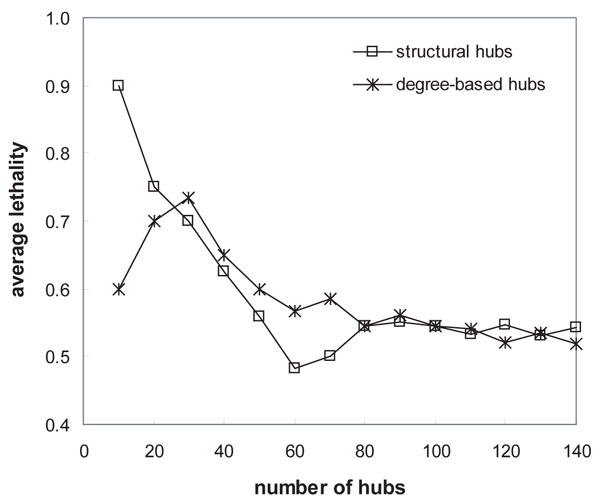
**Biological assessment of structural hubs by protein lethality**. From the list of nodes arranged by their degrees and hub confidence scores in descending order, we observed the proportion of lethal proteins for every 10 nodes. Top 20 structural hubs include more lethal proteins than top 20 degree-based hubs.

We biologically validated the structural hubs by lethality which implies the essentiality for performing function. The lethality has been determined by gene knockout experiments. We obtained the list of lethal proteins from MIPS [27]. In the same way, we enumerated the nodes by degree and hub confidence in a descending order, and monitored the proportion of lethal proteins for every 10 nodes. In Figure 6, we plotted the alteration of cumulative lethality. In contrast to the result of topological assessment, top 20 structural hubs have higher lethality than the same number of degree-based hubs. In particular, the proportion of lethal proteins in top 10 structural hubs is 50% higher than in top 10 degree-based hubs. However, structural hubs in the rank between 50 and 70 have lower lethality than degree-based hubs. It indicates that our structural hub confidence measure ranked highly lethal proteins in top 20, and moved down high-degree but non-lethal proteins to the rank between 50 and 70.

**Figure 7:**
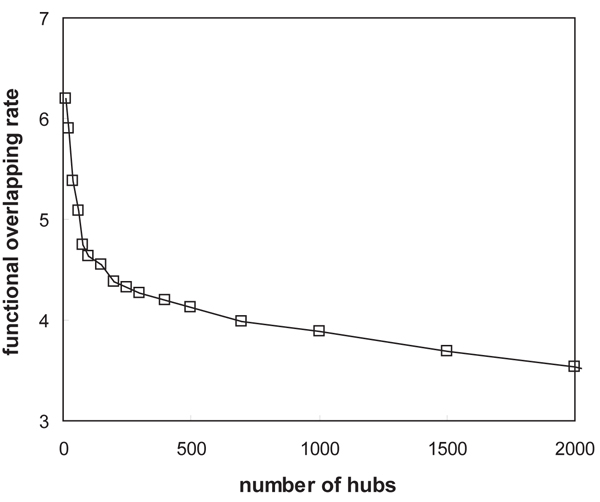
**Average functional overlapping rates of proteins with respect to their hub confidence scores**. We arranged the proteins in the yeast interaction network by their hub confidence scores in descending order, and counted the number of functions that each protein performs. The functional categories on the 3rd level in a hierarchy from MIPS were used. Top 10 structural hubs have an overlapping rate approximately 75% higher than the 2000th protein.

Importantly, most structural hub proteins perform several different functions. We examined functional overlapping rates of the hubs. Among the functional categories in a hierarchy from MIPS, we extracted the ones on the 3rd-level from the top and their annotations. We then inspected how many categories each hub protein appears in. Figure 7 shows the functional overlapping rates of the proteins ordered by hub confidence. The average overlapping rate of 2,000 proteins is around 3.5. However, the rate increases to 4.5 for top 150 structural hubs, and becomes even greater than 6.0 for top 10 structural hubs. This result suggests that, in network topology, structural hubs mostly bridge different functional modules regardless of their degree.

### Modularity of clusters

We implemented clustering of proteins using the tree structure converted from a protein interaction network, and inspected whether the output clusters are likely to be functional modules. Modularity of a sub-network has been commonly estimated by the ratio of the number of edges within the sub-network to the number of all edges starting from the nodes in the sub-network. However, in this estimation, the modularity depends on the number of nodes in the sub-network. For example, suppose a network* G* has 500 nodes. Sub-networks* G′* and* G″* of* G* consist of 10 and 100 nodes, respectively. A node in* G″* has a higher probability having links to the nodes within the same sub-network (intraconnections) and a lower probability having links to the nodes outside of the sub-network (interconnections), comparing to a node in *G′.* We thus normalized the formula of modularity by the probability of a node in the sub-network being linked to the members in the same sub-network.

**Figure 8:**
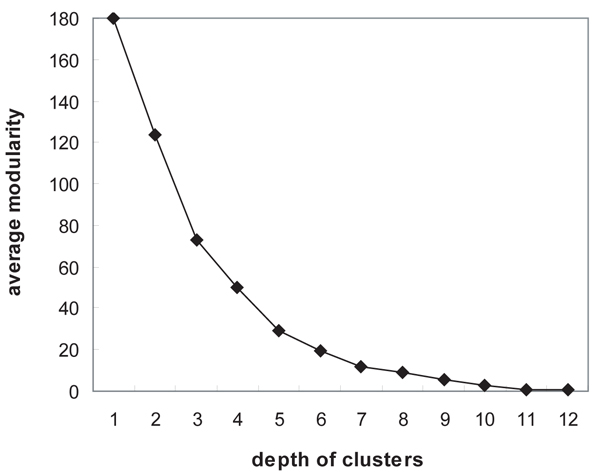
**The normalized modularity pattern with respect to the depth of clusters generated**. Modularity of a sub-network *G′* was calculated by the ratio of the number of edges within *G′* to the number of edges starting from the nodes in *G′*, and it was normalized by the probability of a node in *G′* being linked to the other members in *G′*. As the cluster depth decreases, its normalized modularity has a monotonic increase. It indicates that the modules representing more specific functions have higher modularity.

We grouped the output clusters with regard to their depth, and averaged the normalized modularity for each group. As already remarked in Methods, the depth of a cluster has an inverse relationship with its functional specificity. It is also expected that a more specific functional module in a hierarchy has higher modularity in network topology, i.e., a sub-module* Y* in a module* X* has denser intraconnections than* X*. The experimental result is shown in Figure 8. As the cluster depth decreases, the modularity has a monotonic increase. In particular, it rapidly increases when the depth is less than 6. This result satisfies our expectation of the modularity pattern in a hierarchy. It strongly implies that the hierarchy structured by our approach corresponds to the functional organizations in a protein interaction network. To evaluate clustering accuracy, we used the* f*-measure, which is the harmonic mean of precision and recall. Suppose an output cluster* X* is mapped to an actual functional modules* F_i_.* Recall, which is also called a true positive rate or sensitivity, is the proportion of common members between* X* and* F_i_* to the size of* F_i_.* Precision, which is also called a positive predictive value, is the proportion of common members between* X* and* F_i_* to the size of *X*(14)

For direct comparison of each functional module with clusters in the same level in a hierarchy, the *f*-measure is an appropriate evaluation method since it gives a higher chance to score high when the functional module has the similar size with a cluster. As actual functional modules, we used the annotations on the 2nd-level, 3rd-level and 4th-level functions in a hierarchy from MIPS. Starting from the most general functions on the 1st-level, functions become more specific as the level increases. Then, for each function, we selected a cluster with the best match by* f*-measure. We finally calculated the average *f*-measure across the functions on each level. Table 1 shows the clustering accuracy of our network-conversion approach. For more specific functions, i.e., higher-level functions, we achieved higher accuracy. It indicates that our approach more accurately generated the small-sized clusters for specific functions. Comparing the accuracy of two competing methods of hierarchical clustering: Edge-Betweenness algorithm [16] and ProDistIn [15], our network-conversion approach outperforms the other methods across all levels of functions as shown in Table 1. We additionally evaluated the output clusters comparing to protein complexes from MIPS. The gap of clustering accuracy between our approach and the competing methods becomes even larger.

**Table 1:** Clustering performance comparison by *f* — measure

	Network-conversion	Edge-betweenness	ProDistIn
2nd-level functions	0.326	0.248	0.211
3rd-level functions	0.383	0.247	0.215
4th-level functions	0.438	0.226	0.235
protein complexes	0.425	0.135	0.184

## Conclusions

Decomposing, converting and synthesizing complex systems are fundamental tasks for modeling their structural behavior. Recently, such approaches in protein interaction networks has been widely attempted to understand biological processes and functional organizations within a cell. We have studied the methodology for converting a protein interactome network into an effective structure for the purpose of functional knowledge discovery. For this task, we designed the path strength model and exploited the novel concept of centrality. The generated hierarchical tree structure can be applied to selecting functionally essential hub proteins and identifying functional modules. Unlike other hierarchical clustering methods, our approach dynamically explores the entire hierarchical structure of proteins in a global view. All the individual parent-child relationships between proteins in the hierarchy are meaningful and comparable. The performance of our approach can be more improved by developing the advanced methods, which efficiently integrate a massive amount of current heterogeneous biological data and accurately analyze the reliability of functional associations between interacting proteins.

## Authors contributions

YRC designed and implemented the method, analyzed the results, and drafted the manuscript. AZ coordinated the project, analyzed the results, and revised the final manuscript.

## Competing interests

The authors declare that they have no competing interests.
